# Safeguard Ethics

**Published:** 1916-02

**Authors:** 


### Safeguard Ethics.

Medical ethics is a good thing, and the ethical ideas should be high. There is always danger, however, that harm may be done in the name of medical ethics. Let a member of a profession but suggest that a thing is unethical, and often it is condemned without a proper consideration of whether it is in fact unethical. Medical ethics should never be allowed to stand as a bar to progress in the science of medicine; neither should it be used as an excuse for one physician giving another an undeserved kick. Ethics, because it is such a popular term, is always in need of safeguarding from abuse. It is safe in the hands of those who always behave as gentlemen should behave, but the interpretation is so pliable that it is at times used as a weapon for wrongdoing.

